# The Role of Continuous Monitoring of Venous Drainage Flow and Integrated Oxygen Extraction (ER_i_O_2_) via Bilateral Near-Infrared Spectroscopy in Cerebral Perfusion During Aortic Arch Surgery

**DOI:** 10.3390/medicina61020226

**Published:** 2025-01-27

**Authors:** Ignazio Condello, Giuseppe Speziale, Flavio Fiore, Giuseppe Nasso

**Affiliations:** Department of Cardiac Surgery, Anthea Hospital GVM Care and Research, Via Camillo Rosalba 35/37, 70124 Bari, Italy

**Keywords:** aortic arch surgery, cerebral perfusion, selective antegrade cerebral perfusion, venous drainage flow, oxygen extraction, near-infrared spectroscopy

## Abstract

*Background and Objective:* Effective cerebral perfusion monitoring is essential in aortic arch surgery, particularly when employing the Kazui technique under moderate hypothermia. Near-infrared spectroscopy (NIRS) provides real-time regional oxygen saturation (rSO_2_) measurements, while the continuous monitoring of venous drainage flow and oxygen extraction ratio (ERiO_2_) delivers additional insights into cerebral oxygenation and metabolic balance. This study investigates the correlation between NIRS-derived rSO_2_, venous drainage flow, and ERiO_2_ during selective antegrade cerebral perfusion (SACP) to better understand their interplay and clinical significance. *Materials and Methods:* This retrospective study analyzed data from 10 patients undergoing aortic arch surgery with the Kazui technique, including 4 patients with type I A dissections and 6 with aortic arch aneurysms. Bilateral NIRS (Masimo system) was used to measure rSO_2_, while venous drainage flow and ERiO_2_ were continuously monitored using the Landing system. Intraoperative parameters such as cardiopulmonary bypass (CPB) time, cooling and rewarming duration, venous return flow, and perfusion delivery rates were collected and analyzed. The correlations between rSO_2_, venous drainage flow, and ERiO_2_ were statistically evaluated. *Results:* The mean CPB time was 182 ± 15 min, with a mean cross-clamp time of 98 ± 12 min. Cooling to 20 °C was achieved in 29 ± 3 min, followed by a controlled rewarming phase of 10 ± 1.5 min. The venous return flow averaged 570 ± 25 mL/min, while the perfusion delivery rates exceeded 600 ± 30 mL/min. Bilateral NIRS monitoring revealed stable rSO2 values averaging 65 ± 5%, while ERiO2 averaged 28 ± 4%. A strong correlation (r = 0.91, *p* < 0.01) was observed between rSO_2_ and ERiO_2_, with venous drainage flow playing a critical role in maintaining this relationship. *Conclusions:* This study demonstrates a robust correlation between NIRS-derived rSO_2_, continuous venous drainage flow, and ERiO_2_ during SACP in aortic arch surgery.

## 1. Introduction

Aortic arch surgery, particularly procedures involving selective antegrade cerebral perfusion (SACP) under moderate hypothermia, demands careful management to protect neurological function during periods of circulatory arrest [1,2]. The real-time monitoring of cerebral perfusion has become an essential component of these procedures, with tools like near-infrared spectroscopy (NIRS) providing valuable insights into cerebral oxygenation and metabolic status. NIRS, which measures regional oxygen saturation (rSO_2_), is non-invasive and widely used, but its ability to precisely reflect the balance between oxygen delivery and consumption remains a topic of investigation [3,4]. Recent advancements have introduced the continuous monitoring of venous drainage flow and integrated oxygen extraction (ERiO_2_) as complementary methods to assess cerebral perfusion more comprehensively [5]. Venous drainage flow reflects the adequacy of cerebral outflow, while ERiO_2_ quantifies the proportion of oxygen extracted from the delivered supply, offering a direct measure of metabolic activity [4,5]. The interplay between these parameters and NIRS readings may reveal critical insights into the efficiency of cerebral perfusion strategies. However, the relationship between these variables is not fully understood, and questions remain about the reliability of NIRS as a surrogate marker for cerebral metabolic status in comparison to ERiO_2_ and venous drainage measurements [6,7,8]. Additionally, factors influencing these relationships, such as patient-specific physiology, cannula positioning, and perfusion flow rates, add complexity to their interpretation [9,10,11]. This retrospective study aims to analyze the correlation between NIRS-derived rSO_2_, continuous venous drainage flow, and ERiO_2_ during aortic arch surgery utilizing the Kazui technique. By examining data collected intraoperatively from patients undergoing SACP, this study seeks to explore the interplay among these variables and their potential role in guiding perfusion management. Understanding these relationships could enhance the precision of intraoperative monitoring, improve decision making, and ultimately contribute to better neurological outcomes in complex aortic arch procedures.

## 2. Materials and Methods

**Figure 1:**
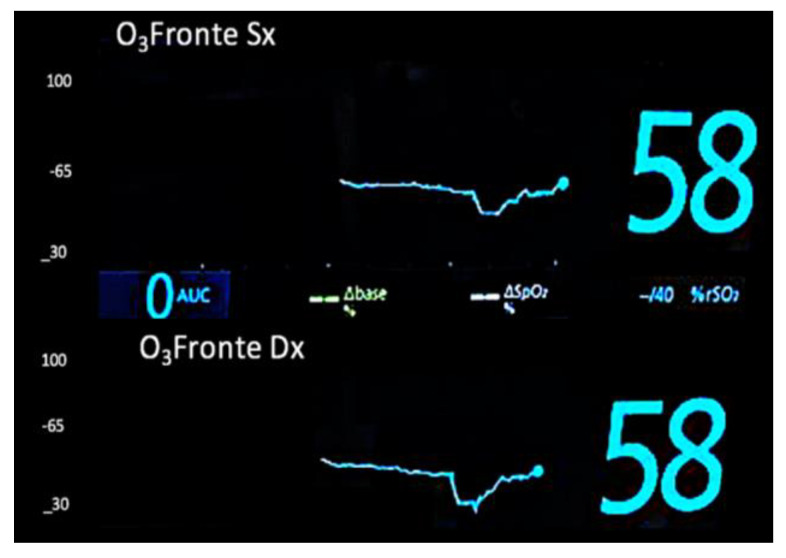
NIRS-derived regional oxygen saturation (rSO_2_) during selective antegrade cerebral perfusion.

**Figure 2:**
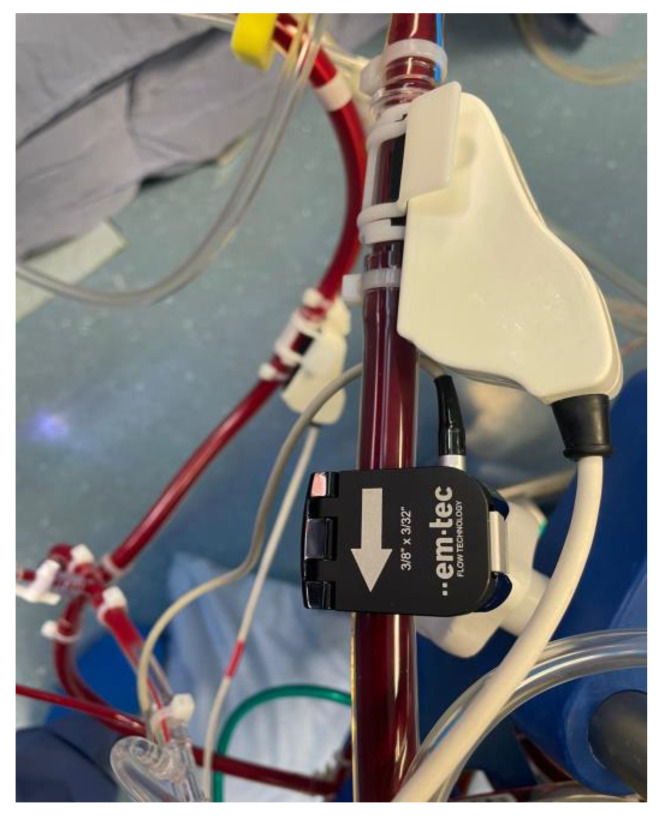
Flowmeter and cuvette in venous drainage line for blood flow monitoring and ERIO_2_ during selective antegrade cerebral perfusion.

**Figure 3:**
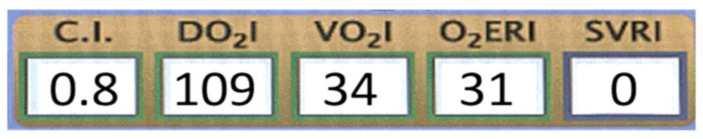
Monitoring of ERiO_2_ and venous blood flow during selective antegrade cerebral perfusion.

This retrospective study analyzed data from ten patients who underwent aortic arch surgery using the Kazui technique for selective antegrade cerebral perfusion (SACP) under profound hypothermia. The study period spanned from November 2022 to November 2024 and included four patients diagnosed with type I A aortic dissections (urgent) and six with aortic arch aneurysms. Surgeries were performed following a standardized protocol designed to optimize cerebral perfusion and minimize neurological risks [1]. Approval was obtained from the Internal Institutional Review Board (IRB), and all protocols complied with the principles outlined in the Declaration of Helsinki. The study adhered strictly to international and institutional guidelines for the ethical conduct of research involving human participants. Due to the retrospective design of the study and the use of de-identified patient data, the requirement for individual informed consent was waived. Surgical procedures were conducted with standard cardiopulmonary bypass (CPB) initiated via peripheral cannulation of the femoral artery (Bio-medicus 19 Fr by Medtronic, Minneapolis, MN, USA) and the femoral vein (23–25 Fr by Livanova, UK), and Custoidol^®^ HTK solution was used for myocardial protection in all procedures. Systemic cooling was performed to achieve a target temperature of 20 °C, monitored through nasopharyngeal and rectal temperature sensors. Upon reaching the target temperature, systemic circulatory arrest was instituted, and bilateral SACP (13 FR manual insufflation cannulas by Medtronic, Minneapolis, MN, USA) was initiated, delivering oxygenated blood at a flow rate calculated as 10 mL/kg based on the patient’s body weight. Perfusion flow was dynamically managed by integrating measured, calculated, and recorded parameters, including right radial arterial pressure, NIRS-derived regional oxygen saturation (rSO_2_) (Masimo), oxygen extraction ratio (ERiO_2_), and venous drainage flow. During cerebral perfusion, continuous monitoring was performed using bilateral near-infrared spectroscopy (NIRS) to assess rSO_2_ (Root^®^ Patient Monitoring by Masimo) (Figure 1), while cerebral venous drainage flow and ERiO_2_ were measured using the Landing monitoring system (Eurosets, Medolla SRL, Italy) (Figure 2 and Figure 3). These parameters were used collectively to guide perfusion adjustments and ensure optimal delivery and metabolic balance during the procedure. Following systemic circulatory arrest and the cerebral perfusion phase, reperfusion was initiated for 10 min at a nasopharyngeal temperature of 20 °C, with blood temperature matching the nasopharyngeal target to minimize thermal gradients. Subsequently, rewarming was carried out, maintaining a temperature gradient of 10 °C between the blood and rectal temperature, until a target core temperature of 36.8 °C was reached. This controlled gradient ensured gradual rewarming and reduced the risk of ischemic–reperfusion injury or excessive thermal stress. All intraoperative parameters, including CPB time, cross-clamp time, cooling and rewarming phases, perfusion pressures, and monitored variables (rSO2, ERiO2, and venous return flow), were recorded. Mean values and standard deviations were calculated for quantitative variables, and Pearson correlation coefficients were used to assess the relationships among NIRS, ERiO_2_, and venous drainage flow. Data analysis was performed using SPSS Statistics Version 28.0, with statistical significance set at *p* < 0.05. This analysis aimed to evaluate the interplay between continuous cerebral monitoring parameters and their correlation with optimal cerebral perfusion during SACP, with secondary outcomes, including postoperative neurological status, assessed using standardized evaluation protocols.

## 3. Results

**Figure 4:**
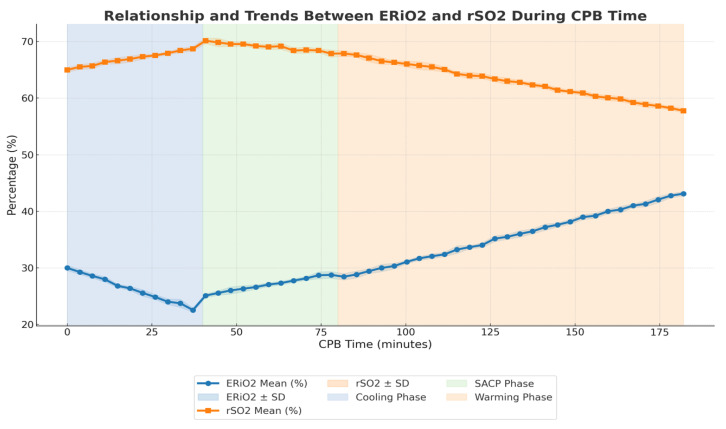
Relationship and trend between ERiO2 and rSO2 during CPB time.

This study included a total of 10 patients who underwent aortic arch surgery using the Kazui technique under profound hypothermia. The cohort consisted of four patients with type A aortic dissections and six with aortic arch aneurysms (Table 1). Intraoperative data revealed that the mean cardiopulmonary bypass (CPB) time was 182 ± 15 min, and the mean cross-clamp time was 98 ± 12 min. The cooling phase to achieve a target temperature of 20 °C lasted 29 ± 3 min, followed by a reperfusion phase at 20 °C for 10 ± 1.5 min. Rewarming was completed in 40 ± 5 min, with a controlled gradient of 10 °C between the blood and rectal temperature, reaching a final core temperature of 36.8 °C. The bilateral selective antegrade cerebral perfusion (SACP) flow rates averaged 620 ± 30 mL/min, while the venous return flow averaged 570 ± 25 mL/min. The cerebral perfusion time during SACP was 40 ± 6 min, and the circulatory arrest time was 42 ± 7 min. Continuous cerebral monitoring showed stable bilateral NIRS-derived regional oxygen saturation (rSO2) values, averaging 65 ± 5%**,** and a mean oxygen extraction ratio (ERiO2) of 28 ± 4%**.** During the cooling phase, a reduction in oxygen extraction was observed, while, during the warming phase, the ERiO2 increased significantly. This dynamic behavior highlighted the responsiveness of cerebral oxygenation to temperature modulation (Table 2). A strong correlation was observed between rSO2 and ERiO2 (r = 0.91, *p* < 0.01), underscoring the consistency between cerebral oxygenation and metabolic activity during perfusion (Figure 4).

Postoperatively, all patients demonstrated favorable neurological outcomes, with no cases of stroke, transient ischemic attacks, or major neurological deficits. The mean duration of mechanical ventilation was 8.5 ± 2.3 h, the mean intensive care unit (ICU) stay was 48 ± 12 h, and the mean hospital stay was 10 ± 3 days. No significant complications related to the perfusion strategy were observed, and the 30-day mortality rate was 0% (Table 3).

## 4. Discussion

This study highlights the feasibility and effectiveness of integrating near-infrared spectroscopy (NIRS), oxygen extraction ratio (ERiO2), and continuous venous drainage flow monitoring to optimize cerebral perfusion during aortic arch surgery using the Kazui technique [1,2,3,4,5,6,7,9]. The observed correlation between rSO2 and ERiO2 across the phases of cardiopulmonary bypass (CPB) offers valuable insights into the dynamics of cerebral oxygenation and metabolism under profound hypothermia [10,11]. By offering a more comprehensive assessment of cerebral perfusion, this approach could be pivotal in minimizing neurological complications and optimizing surgical results. Despite these promising findings, several limitations must be acknowledged when interpreting the results. First, the small sample size of ten patients limits the generalizability of the results, necessitating larger multicenter studies to validate these findings. The single-center nature of the study introduces potential bias, as the results may be influenced by standardized institutional protocols, which might not be representative of broader practices. Furthermore, the retrospective design is inherently limited by the absence of randomization and the inability to control for all potential confounders. Unmeasured variables, such as individual patient comorbidities or slight differences in surgical technique, may have influenced the outcomes. Additionally, this study focuses exclusively on short-term outcomes, such as neurological status, ICU, and hospital stay durations, and does not address long-term follow-up or the durability of these favorable results. Moreover, the absence of a control group or comparative cohort prevents definitive conclusions about the superiority of this monitoring approach over other strategies. The accuracy of NIRS measurements can be affected by extracranial contamination or variations in systemic factors like hemoglobin levels and the accuracy of ERiO2 under varying conditions. Similarly, ERiO2 interpretation depends on multiple factors, including perfusion pressure and flow dynamics, which were not fully explored in this study. While profound hypothermia is widely accepted as a strategy for cerebral protection during aortic arch surgery, it carries inherent risks, such as coagulopathy and prolonged CPB times, which were not specifically evaluated for their impact on the observed outcomes. Future efforts should focus on conducting larger multicenter prospective trials, incorporating control or comparative groups, and performing long-term follow-up to assess the sustainability of these outcomes. Investigating the interplay between NIRS, ERiO2, and systemic parameters, such as blood pressure, hemoglobin levels, and CPB flows, could refine perfusion protocols. Additionally, advanced analytical approaches, such as machine learning, could leverage real-time data from NIRS and ERiO2 to develop predictive models for optimizing cerebral perfusion strategies [8]. This study underscores the importance of integrating NIRS, ERiO2, and venous drainage flow monitoring in managing cerebral perfusion during complex aortic surgeries. While the findings are promising, further research is essential to address the limitations and refine strategies for achieving optimal neurological outcomes.

## 5. Conclusions

This preliminary study provides insights into the potential benefits of integrating the continuous monitoring of venous drainage flow, oxygen extraction ratio (ERiO2), and NIRS-derived regional oxygen saturation (rSO2) during aortic arch surgery using the Kazui technique. Our observations indicate a correlation between rSO2 and ERiO2, suggesting that these parameters may be complementary in assessing cerebral oxygenation and metabolic balance. Venous drainage flow also appears to play a crucial role in maintaining cerebral perfusion and oxygen delivery. While these findings are encouraging, it is important to acknowledge that they are based on a limited dataset from a single-center study with a small sample size. Therefore, conclusions regarding the efficacy and safety of the monitoring approach should be viewed with caution. The data do not conclusively prove the superiority of this technique over others, but rather point to potential areas where such integrated monitoring could enhance real-time decision making and surgical outcomes. Future research should focus on validating these preliminary results in a larger and more diverse cohort. Expanding the study to include multicenter data would help in assessing the reproducibility and generalizability of the findings. Moreover, exploring long-term outcomes and integrating advanced predictive models could provide deeper insights into refining cerebral perfusion strategies during complex cardiovascular procedures. This approach would further substantiate the role of continuous monitoring modalities in improving patient care in aortic arch surgeries.

## Figures and Tables

**Table 1:** Preoperative data.

Variable	Value
Sample Size (n)	10
Mean Body Surface Area (BSA, m^2^)	1.8 ± 0.1
Mean BMI (kg/m^2^)	26.5 ± 3.2
Mean EuroSCORE	7.8 ± 2.5
Type A Dissections (urgent, n)	4
Aortic Arch Aneurysms (n)	6
Mean Age (years)	64.3 ± 7.8
Male (n, %)	6, 60%
Hypertension (n, %)	8, 80%
Diabetes (n, %)	2, 20%
History of Smoking (n, %)	5, 50%
Mean LVEF (%)	55% ± 5%
Preoperative Neurological Deficits (n, %)	0, None

The data are expressed as %, numerical values, and mean ± standard deviations. BSA: body surface area; BMI: body mass index; EuroSCORE: European System for Cardiac Operative Risk Evaluation; and LVEF: left ventricular ejection fraction.

**Table 2:** Perioperative data.

Variable	Value
CPB Time (minutes)	182 ± 15
Cross-Clamp Time (minutes)	98 ± 12
Cooling Phase Duration (minutes)	29 ± 3
Reperfusion Phase Duration (minutes)	10 ± 1.5
Rewarming Phase Duration (minutes)	40 ± 5
Cerebral Perfusion Time (minutes)	40 ± 6
Circulatory Arrest Time (minutes)	42 ± 7
Target Core Temperature (°C)	36.8
SACP Flow Rate (mL/min)	620 ± 30
Venous Return Flow on SACP (mL/min)	570 ± 25
rSO2 SACP (%)	65 ± 5%
ERiO2 SACP (%)	28 ± 4%
Hemoglobin (Hb, g/dL)	9.5 ± 1.0
Flow During Cooling (L/min)	4.00 ± 0.14
Flow During Warming (L/min)	5.00 ± 0.14
DO2i During Cooling (mL/min/m^2^)	320 ± 30
DO2i During Warming (mL/min/m^2^)	350 ± 35
Mean Arterial Pressure (mmHg)	65 ± 5
Transfusion Rate (units/patient)	1.2 ± 0.3

The data are expressed as numerical values and mean ± standard deviations. CPB: cardiopulmonary bypass; SACP: selective antegrade cerebral perfusion; rSO2: regional oxygen saturation; ERiO2: indexed oxygen extraction ratio; Hb: hemoglobin; and DO2i: indexed oxygen delivery.

**Table 3:** Postoperative data.

Variable	Value
Neurological Deficits	None
Mechanical Ventilation Duration (hours)	8.5 ± 2.3
ICU Stay Duration (hours)	48 ± 12
Hospital Stay Duration (days)	10 ± 3
30-day Mortality Rate (%)	0%

The data are expressed as %, numerical values, and mean ± standard deviations. ICU: intensive care unit.

## Data Availability

The data presented in this study are available upon request from the corresponding author.

## References

[1] Braverman A.C. (2010). Acute aortic dissection: Clinician update. Circulation.

[2] Di Mauro M., Iacò A.L., Di Lorenzo C., Gagliardi M., Varone E., Al Amri H., Calafiore A.M. (2013). Cold reperfusion before rewarming reduces neurological events after deep hypothermic circulatory arrest. Eur. J. Cardiothorac. Surg..

[3] Yujian Y., Juan L., Peiyun Z., Yaoguang F., Khan A., Zhengwen L. (2024). Application of Bilateral Cerebral Perfusion + Balloon Occlusion of Descending Aorta + Antegrade Perfusion of Lower Body in Debakey Type I Aortic Dissection. J. Coll. Physicians Surg. Pak..

[4] Condello I., Montemurro V., De Rosis M.G., Nasso G. (2024). Selective monitoring of superior and inferior vena cava drainage flows in bicaval cannulation: Potential clinical benefits of proximal junction placement on the CPB side. Perfusion.

[5] Kazui T. (2013). Total arch replacement with separated graft technique and selective antegrade cerebral perfusion. Ann. Cardiothorac. Surg..

[6] Abdulwahab H.A.M., Kolashov A., Haneya A., Klump H., Moza A., Arab M.F., Shoaib M., Zayat R., Khattab M.A. (2024). Temperature management in acute type A aortic dissection treatment: Deep vs. moderate hypothermic circulatory arrest. Is colder better?. Front. Cardiovasc. Med..

[7] Langenhorst J., Benkert A., Peterss S., Feuerecker M., Scheiermann T., Scheiermann P., Witte M., Benkert A., Bayer A., Prueckner S. (2024). Agreement of in-ear temperature to core body temperature measures during invasive whole-body cooling for hypothermic circulatory arrest in aortic arch surgery. Sci. Rep..

[8] Peng Q., Cai M., Chen X., Lin T., Meng W., Guan L., Zhu P., Zheng S., Lu J., Zhou P. (2023). Nadir oxygen delivery during cardiopulmonary bypass in acute type A aortic dissection repair. J. Thorac. Dis..

[9] Luehr M., Bachet J., Mohr F.W., Etz C.D. (2014). Modern temperature management in aortic arch surgery: The dilemma of moderate hypothermia. Eur. J. Cardiothorac. Surg..

[10] Berger T., Rylski B., Czerny M., Kreibich M. (2023). Selective antegrade cerebral perfusion: How to perfuse?. Eur. J. Cardiothorac. Surg..

[11] Friess J.O., Beeler M., Yildiz M., Guensch D.P., Levis A., Gerber D., Wollborn J., Jenni H., Huber M., Schönhoff F. (2023). Determination of selective antegrade perfusion flow rate in aortic arch surgery to restore baseline cerebral near-infrared spectroscopy values: A single-centre observational study. Eur. J. Cardiothorac. Surg..

